# Editorial: Imaging the developing connectome of perinatal brain

**DOI:** 10.3389/fnins.2023.1122829

**Published:** 2023-02-06

**Authors:** Dan Wu, Weihao Zheng, Patricia Ellen Grant, Hao Huang

**Affiliations:** ^1^Key Laboratory for Biomedical Engineering of Ministry of Education, College of Biomedical Engineering and Instrument Science, Zhejiang University, Hangzhou, China; ^2^Gansu Provincial Key Laboratory for Wearable Computing, School of Information Science and Engineering, Lanzhou University, Lanzhou, China; ^3^Fetal Neonatal Neuroimaging and Developmental Science Center, Boston Children's Hospital, Boston, MA, United States; ^4^Division of Newborn Medicine, Boston Children's Hospital, Boston, MA, United States; ^5^Department of Radiology, Boston Children's Hospital, Boston, MA, United States; ^6^Department of Pediatrics, Harvard Medical School, Boston, MA, United States; ^7^Department of Radiology, Children's Hospital of Philadelphia, Philadelphia, PA, United States; ^8^Department of Radiology, Perelman School of Medicine, University of Pennsylvania, Philadelphia, PA, United States

**Keywords:** magnetic resonance imaging (MRI), perinatal brain, connectome, imaging biomarkers, development, analytic methods

Brain maturation during the perinatal period in the fetus and infant is a rapid and complex process. Neurodevelopment during this period is critical for supporting later cognitive, emotional, and behavioral abilities. Increasing evidence for the perinatal origins of various neurodevelopmental disorders underscores the importance of identifying features of early brain development (Dehaene-Lambertz and Spelke, 2015). Understanding the developing brain connectome will open new insights into the fundamental processes of brain circuit formation and maturation in early life and reveal the etiology of intractable neurodevelopmental disorders. Advances in magnetic resonance imaging (MRI), such as rapid imaging and motion correction techniques, have overcome significant challenges in fetal and infant brain MRI and enabled non-invasive *in vivo* assessment of functional and structural connectivity between separate brain regions (Kaiser, 2017; Dubois et al., 2021), offering great opportunities to capture the connectome of the fetal and postnatal brain with unprecedented accuracy. Thus, the purpose of this Research Topic focuses on neuroimaging studies of the early development of the brain connectome.

This Research Topic includes 8 original research paper and 1 data descriptor. Main research contents concentrate on atypical connectome pattern and novel imaging biomarkers for prematurity, hypoxic ischemic encephalopathy (HIE), etc. and machine learning algorithms for fetal brain analysis. Neumane et al. explored the impact of prematurity on the development and integrity of the sensorimotor connectivity and their relationship to later motor impairments. They found that prematurity affected early microstructural development of the primary sensorimotor network and these effects differed according to the level of prematurity. They also highlighted the microstructural development of specific tracts predicted fine motor and cognitive outcomes at 18 months. Li et al. investigated the effects of daily iron supplementation on motor development and brain structural connectivity of preterm infants. They found that iron status at early postnatal period was related to both motor development and connectivity decreases, and the delayed motor development can be reversed by supplying 2 mg/kg of iron per day for 6 months. Vishnubhotla et al. studied the influence of prenatal opioid exposure on brain structural connectivity, and identified two connectivity pathways that were significantly differed between opioid exposure infants and unexposed controls. Votava-Smith et al. reported that clinical risk factors and brain dysplasia score were associated with distinct brain dysmaturation patterns in term neonates with congenital heart disease (CHD). Specifically, clinical factors were most predictive to postnatal microstructural dysmaturation, whereas subcortical dysplasia predicted connectome-based measures, suggesting the complementary effects of connectome and microstructure in deciphering risk factors related to poor neurodevelopment in CHD. Based on the least absolute shrinkage and selection operator (LASSO) regression model, Onda et al. developed a novel biomarker named composite diffusion tensor imaging (cDTI) score to assess the severity of short-term neurological functions of HIE neonates. They demonstrated high cDTI scores were related to the intensity of the early inflammatory response and the severity of neuronal impairment after therapeutic hypothermia.

Characterizing fetal brain development *in utero* is still challenging due to the difficulties in acquiring high-quality MRI data and lack of effective analytic methods. Based on 188 brain MRI of normal fetuses ranging from 19 to 37 gestational weeks, Ren et al. establish a reference of intracranial structure volumes during this period by manual segmentation from two experienced experts. Wang et al. developed a MRI-based semi-automatic pipeline to segment the cortex and subcortical structures of fetal brains, reducing the costs of manual segmentation. De Asis-Cruz et al. proposed a full automatic and computationally efficient generative adversarial neural network for segmenting the fetal brain based on functional MRI, which yielded whole brain masks that closely approximated the manually labeled ground truth. This study is of great significance in facilitating *in utero* investigations of emerging functional connectivity.

Lack of available and reliable data is one of dominating factors that limits the exploration of brain maturational trajectories early in life. Edwards et al. introduced the neonatal data release of the Developing Human Connectome Project, which includes 887 multimodal high-quality MR images from 783 preterm-born and term-born infants and essential metadata. This open dataset allows researchers to design the experiment as they wish, making great contribution to uncover the typical and atypical brain development across the perinatal period.

In conclusion, these nine papers included in this Research Topic summary the recent progression of normal brain maturation and markers of neurodevelopmental disorders during the perinatal period, as well as important technical advances in fetal and infantile brain MRI.

## Author contributions

All authors listed have made a substantial, direct, and intellectual contribution to the work and approved it for publication.
